# Editorial: Novel insights on the role of bacterial membrane proteins in virulence and pathogenesis

**DOI:** 10.3389/fcimb.2023.1282672

**Published:** 2023-09-07

**Authors:** Harapriya Mohapatra, Jesús Arenas

**Affiliations:** ^1^School of Biological Sciences, National Institute of Science Education and Research, Jatani, India; ^2^Homi Bhabha National Institute, BARC Training School Complex, Mumbai, India; ^3^Unit of Microbiology and Immunology, University of Zaragoza, Faculty of Veterinary, Zaragoza, Spain; ^4^Institute Agrofood of Aragón-IA2, University of Zaragoza-Centro de Investigación y Tecnología Agroalimentaria de Aragón (CITA), Zaragoza, Spain

**Keywords:** infection, membrane proteins, virulence, pathogenesis, host-pathogen interaction

In 1884, Christian Gram’s staining revealed the differential nature of cell envelopes in two classes of bacteria. Almost a decade later Glauert and Thornley (1969) using an electron -microscopy technique evidenced a distinct two-layered structure of Gram-negative bacteria versus a thick layer of Gram-positive bacteria. Later Miura and Mizushima (1968) and Osborn et al. (1972) developed biochemical methods to characterize cytoplasmatic and outer membrane layers. Bacterial membranes are not only important to maintain cell integrity and regulate the import of nutrients/antimicrobials but, also play relevant functions in pathogenesis functioning as a platform to expose a repertoire of virulence factors. These factors include associated or integral membrane proteins, as those studied in this Research Topic. Besides, the discovery of membrane vesicles reiterated the significant role of the composition of both membranes in virulence and pathogenesis. With this background, the current thematic issue was aimed to invite novel findings on how bacterial membrane proteins facilitate and/or modulate pathogenesis. At the end, five articles were edited; four of which are original research articles. Two articles report novel functions of cell surface collagen binding adhesins in Gram-positive pathogens *Streptococcus* sp while the other two unveil the role of outer membrane proteins in the Gram-negative pathogen *Haemophilus influenzae*.

Original research by Naka et al., elucidated the role of collagen-binding protein Cnm of *Streptococcus mutans*. Investigations of the Cnm-deficient isogenic mutant strains showed that indeed, Cnm is a relevant protein for collagen binding in this bacterium. Diverse electron microscopy assays showed that the protein is located around the bacteria cell wall and produces protrusions at the membrane surface. Comparative RNA-seq assays evidenced that the lack of Cnm production altered the expression of several genes including biofilm formation-associated genes, and, indeed, biofilm formation assays evidenced that Cnm contributes to biofilm formation in the absence of collagen. *Streptococcus suis* encodes a collagen-binding protein called Cba. By exploring the phenotypes of a Cba-depletion mutant, authors demonstrated that Cba has implications in biofilm formation and phagocytic resistance to macrophages with relevant implications in virulence as revealed by an attenuated phenotype in mice infection models. Immunization of mice with purified protein induced strong antibody responses, but resulted in no protection and caused organ lessons and mortality emulating a phenomenon called antibody-dependent enhancement of infection, previously described for other proteins (Halstead et al., 2010).

The cell wall is an interconnected matrix of peptidoglycan that surrounds the cytoplasmic membrane and protects the cell from osmotic lysis. Peptidoglycan biosynthesis comprises the synthesis of precursors in the cytoplasm, their transport to the outer face of the cytoplasmic membrane, and their subsequent cross-linking into the existing peptidoglycan layer. The latter process is mediated by enzymes with peptidoglycan-glycosyl transferase and transpeptidase activities. The most studied enzymes are penicillin-binding proteins (PBPs), which are classified as PBP type A and PBP type B. Early work showed that the activity of some PBPs is regulated by lipoproteins, i.e. LpoA and LpoB. Both proteins activate transpeptidase and the peptidoglycan polymerization of PBP1a and PBP1b, respectively (Sardis et al., 2021). A research article by Jalalvand et al. investigated the role of LpoA in the formation of outer membrane vesicles (OMVs) in the respiratory tract pathogen *Haemophilus influenzae* strain 3655. Fluorescence microscopy assays revealed that LipoA accumulated in OMVs and was located in the septum during cell division. Through reporter fusion protein studies, the authors have unveiled that the C-terminal domain of the protein is required for its transportation to OMVs.

Further, in a brief report Su et al., investigated the outer membrane protein of P5 from non-typeable *Haemophilus influenzae*. P5 is structured in a β-barrel transmembrane domain that contains four extracellular loops and a C-terminal domain (Novotny and Bakaletz, 2003). The authors showed that P5 binds to the peptidoglycan layer. Deletion of P5 generated a viable mutant that exhibited a reduced abundance of virulence factors in the outer membrane and periplasmic chaperones. Depletion of P5 caused a variety of phenotypes including, i) reduced adherence to cells, ii) reduced binding to fibronectin, iii) reduced survival to complement mediate killing, and iv) enhanced OMV production. This effect was majorly caused by the depletion of its C-terminal domain.

The development of new ideas and strategies to counter bacterial pathogens is of utmost importance in this post-antibiotic era, where unprecedented escalation of drug-resistant organisms is being observed. Capsular vaccines and bacterins gained attention as these function by stimulating immune response. The perspective by Ewasechko et al., provides insights from the study of transferring binding proteins of human and animal pathogens with demonstrated potential in vaccine development. The authors also speculate that the presence of these receptors in pathogenic bacteria of the upper respiratory tract of human and porcine pathogens could be related to the differential expression of transferrin between the subepithelial space and the air-liquid interface that varies iron availability. Therefore, transferrin-binding proteins could contribute to iron acquisition during invasion. This is supported by the presence of these proteins in human and porcine pathogens and their lack of commensal species.

All together, we hope this Research Topic provides valuable insights into the role of bacterial membranes in virulence and pathogenesis and the development of novel vaccines.

## Author contributions

HM: Writing – original draft, Writing – review & editing. JA: Writing – original draft, Writing – review & editing.
